# Defining cognitive and functional profiles in schizophrenia and affective disorders

**DOI:** 10.1186/s12888-020-2459-y

**Published:** 2020-01-31

**Authors:** Yu-Chi Huang, Yu Lee, Chun-Yi Lee, Pao-Yen Lin, Chi-Fa Hung, Sheng-Yu Lee, Liang-Jen Wang

**Affiliations:** 1 Department of Psychiatry, Kaohsiung Chang Gung Memorial Hospital and Chang Gung University College of Medicine, Kaohsiung, Taiwan; 2 Department of Psychiatry, Kaohsiung Veterans General Hospital, Kaohsiung, Taiwan; 3 Department of Psychiatry, College of Medicine, National Yang-Ming University, Taipei, Taiwan; 4 Department of Psychiatry, School of Medicine, and Graduate Institute of Medicine, College of Medicine, Kaohsiung Medical University, Kaohsiung, Taiwan; 5 Department of Child and Adolescent Psychiatry, Kaohsiung Chang Gung Memorial Hospital and Chang Gung University College of Medicine, No.123, Ta-Pei Road, Kaohsiung, Taiwan

**Keywords:** Neurocognitive tests, Schizophrenia, Depression, Mania, Daily skill functioning

## Abstract

**Background:**

Neurocognitive dysfunction is a common symptom of various major psychiatric disorders, including schizophrenia, major depressive disorder (MDD), and bipolar I disorder (BD). In this study, we investigated whether cognitive profiles and daily skill functioning could effectively differentiate between patients with schizophrenia, MDD, and BD.

**Method:**

In this cross-sectional study, we recruited a total of 63 patients with schizophrenia, 55 patients with MDD, 43 patients with BD, and 92 healthy control subjects. We evaluated participants’ cognitive functions and functional capacity using the Brief Assessment of Cognition in Schizophrenia (BACS) and the UCSD Performance-based Skills Assessment, Brief Version (UPSA-B), respectively. Multivariate analysis of covariance was then adopted to determine inter-group differences in BACS and UPSA-B performance.

**Results:**

The BACS was capable of differentiating patients with a major psychiatric disorder (schizophrenia, MDD, and BD) from healthy subjects. Furthermore, schizophrenia patients had poorer motor speed performance than patients with affective disorders. The UPSA-B, particularly the financial portion, was able to distinguish schizophrenia patients from other groups. However, we did not observe any differences in UPSA-B performance between patients with mood disorders and the healthy controls. No significant difference between patients with BD and those with MDD were observed in either cognitive function or in functional capacity. The performances of the BACS and the UPSA-B were positively correlated, particularly in the MDD group.

**Conclusion:**

Considering overall performance, the BACS and the UPSA-B characterize different endophenotyping profiles in the aforementioned four participant groups. Therefore, the results support the need for comprehensive assessments that target both cognitive function and functional capacity for patients with major psychiatric disorders.

## Background

Neurocognitive dysfunction is a common symptom of various major psychiatric disorders, including schizophrenia [1], bipolar I disorder (BD), and major depressive disorder (MDD) [2, 3]. For example, patients with schizophrenia may have cognitive impairments in such domains as attention, motor and processing speed, verbal and spatial memory, working memory, and executive function [4, 5]. Meanwhile, patients with MDD and BD demonstrate comparable cognitive deficits in attention, processing speed, episodic memory, and executive function [6, 7]. Therefore, cognitive ability profiles may potentially serve as a candidate for intermediate endophenotype for recognizing the underlying pathogenesis [8–10] among commonly overlapping symptomatology in patients with psychotic disorders [11, 12]. Cognition impairment significantly worsens patients’ ability to function in daily life, as well as their long-term treatment outcomes [13–15]. Establishing a cognitive battery of tests capable of effectively distinguishing between patients with schizophrenia, MDD, and BD and evaluating cognitive performance during treatment in clinical practice is crucial.

The Brief Assessment of Cognition in Schizophrenia (BACS) was developed as a brief battery of tests to assess cognition to potentially contribute to researchers of clinical trials prior to developing the Measurement and Treatment Research to Improve Cognition in Schizophrenia (MATRICS) [16]. The selected cognition battery subtests were the six cognitive function domains that are considerably impaired in schizophrenia (i.e., verbal memory, motor speed, working memory, verbal learning, attention, and executive function) [17] and significantly associated with real-world functional outcomes in schizophrenia patients [18, 19]. The BACS is a straightforward test and can be administered by different specialties in about 30–35 min [20]. Furthermore, the Chinese BACS has been validated as a reliable cognitive assessment tool [21]. Several studies have used BACS to differentiate patients with major psychiatric diseases, with mixed results. For example, one study [22] indicated that the BACS serves as a practically instructive tool for evaluating the cognitive function of elderly individuals with BD. Another study [23] suggested that BD and schizophrenia presented with similar impairments in neurocognitive functioning, while MDD patients expressed fewer neurocognitive impairments compared to either BD or schizophrenia. However, Chen et al. [24] indicated that more severe deficits in certain cognitive domains were found in patients with schizophrenia compared to those with BD.

Although the literature on cognitive deficits in patients with psychosis- or mood-spectrum disorders has been growing, evidence focusing on evaluating patients’ functional impairments and disabilities and their associations with cognitive function is still lacking. Regarding the research focusing on schizophrenia patients, neurocognition only accounts for a moderate association with variance in functional outcome [25]. The battery for evaluating disability in major mentally ill individuals has differentiated measures of daily adaptive capacity from real-world functional outcomes [26]. The University of California, San Diego (UCSD) Performance-based Skills Assessment, Brief Version (UPSA-B), a brief version of the UCSD Performance-Based Skills Assessment (UPSA) [27], was developed to measure daily functioning in individuals with mental disorders [28]. The UPSA provides a brief measure of functional capacity and can predict a schizophrenia patient’s ability to live independently in society [29, 30]. The UPSA-B has also been effectively used to measure capacity for daily skill functioning in patients with schizophrenia and BD [31]. When adopting the UPSA-B as the major battery to evaluate functional capacity, the performance of patients with mood-spectrum disorder generally surpassed the performance of those with psychosis-spectrum disorder [32]. The aforementioned findings indicate that performance-based measures of daily skills for living are sensitive to detecting and distinguishing the influence of the manifestation of major psychiatric disorders [33].

No study has yet explored the distinguishable performance of the BACS and UPSA-B among patients with schizophrenia, MDD, and BD. Therefore, to fill the research gap, we investigated the performance of cognitive profiles and daily skill functioning of patients with schizophrenia, MDD, and BD by applying the BACS and UPSA-B, respectively. Relevant correlations were simultaneously compared between BACS and the three domains of UPSA-B across the diagnoses of interest. We aimed to determine the different levels of performing cognitive function and functional capacity and their associations between the aforementioned three patient groups and the healthy controls.

## Method

### Study participants

We recruited patients with schizophrenia, BD, or MDD from the out-patient-department, acute ward, or day-treatment center of Kaohsiung Chang Gung Memorial Hospital (KCGMH). The inclusion criteria for patients were as follows: (a) diagnosis of schizophrenia, BD, or MDD in accordance with the criteria of the Diagnostic and Statistical Manual of Mental Disorders, Fourth Edition (DSM-IV-TR) [34]; (b) age ≥ 18 years; (c) without any known systemic or neurological diseases that may confound cognitive performance; and (d) ability to speak Mandarin and read Chinese and provide informed consent. We ultimately recruited 63 patients with schizophrenia, 55 patients with MDD, and 43 patients with BD. We interviewed the patient groups and performed the batteries of neuropsychological tests when the patients’ symptoms had been relatively stable for at least 1 week (the total scores of the Positive and Negative Syndrome Scale (PANSS) were less than 95 for schizophrenia patients [35]; scores of the 17-item Hamilton Depression Rating Scale (17-item HAM-D) were less than 17 for MDD patients [36]; and scores of the Young Mania Rating Scale (YMRS) were less than 26 for BD patients [37]).

We recruited the healthy control group from volunteers from Kaohsiung City and KCGMH personnel. The inclusion criteria consisted of the following: (a) without a history of major psychiatric disorders (psychosis, mood disorders, dementia, organic mental disorders) or systemic or neurological diseases that may potentially cause a cognitive performance bias; (b) age ≥ 18 years; (c) with no first-degree relative with a history of schizophrenia; and (d) ability to speak Mandarin and read Chinese and provide informed consent. We recruited a total of 98 healthy control subjects. Six KCGMH staff who had experience with psychometric testing were excluded from the analyses, while the remaining 92 healthy controls had no experience with psychometric testing.

### Assessment of cognitive profile: the brief assessment of cognition in schizophrenia (BACS)

We evaluated the cognitive functions of all participants using the Brief Assessment of Cognition in Schizophrenia (BACS) [17], which is a battery of cognition tests that measure the cognition domains that have the greatest deficits and correlate significantly with those of real-world functioning in schizophrenia patients [19]. The BACS is generally administered in approximately 30–35 min, and the efficiency generates both a high completion rate and high test–retest reliability. The BACS battery serves as a neuropsychological assessment scheme for patients with various psychosis-spectrum disorders [38]. The BACS consists of seven subtests: the List Learning Test, Digit Sequencing Task, Token Motor Task, Category Instances Test, Controlled Oral Word Association Test, Symbol Coding, and Tower of London Test. These subtests measure verbal memory, working memory, motor speed, verbal fluency, attention and processing speed, and executive function, respectively [20]. The Chinese version of the BACS has been created and proven to have satisfactory reliability and validity [21], and our research team has demonstrated the normative data [39].

### Assessment of functional profile: the UCSD performance-based skills assessment, brief version (UPSA-B)

The UPSA-B, a modified brief version of the UPSA, was developed to evaluate daily functioning in individuals with mental disorders [28]. The UPSA-B consists of two subtests: the financial portion and the communication domain. In the financial portion, participants are required to count out specific amounts of real currency, make change, and request a bank check to pay a bill. Meanwhile, the communication domain asks participants to correctly call directory assistance to obtain a telephone number to reschedule an appointment in a hospital. Three sub-scores are derived from the UPSA-B: Financial skill 1 (counting money and making change), Financial skill 2 (paying a bill), and Communication skill (dialing a telephone number and calling to reschedule an appointment) [40]. The validity of the Chinese version of the UPSA-B has previously been established in Mandarin-speaking patients with mental illnesses [33].

### Psychopathological assessment

Patients with schizophrenia were assessed using the PANSS, which contains 30 items rated on a 7-point Likert scale, with higher scores indicating more severe psychotic symptoms [41]. We calculated subscale scores based on the subsets of three domains: positive, negative, and general psychopathological symptoms [42]. In contrast, patients with MDD or BD were evaluated using the 17-item HAM-D for measuring psychopathology. Patients with BD were also assessed using the YMRS. The 17-item HAM-D includes 17 items for clinicians to rate the severity of depressive symptoms [43], with higher scores representing more severe depression [44]. The YMRS is frequently used to evaluate the severity of manic symptoms [45]. The total score can range from 0 to 60, with higher scores indicating a greater severity of manic symptoms.

### Psychotropic drugs

Any psychotropic drugs being used were recorded, including antidepressants, antipsychotics, benzodiazepines, and mood stabilizers. Agonist activity at acetylcholine muscarinic type 1 (M1) receptors has been demonstrated to enhance memory and learning in schizophrenia [46]. Based on the properties of molecular targets [47], antipsychotics were categorized into the high muscarinic-binding affinity group (Clozapine or Olanzapine) and the low muscarinic-binding affinity group (antipsychotics other than Clozapine or Olanzapine). The dose of antipsychotic drugs was re-calculated based on the defined daily dose recommended by the WHO Collaborating Centre for Drug Statistics Methodology (http://www.whocc.no/atc_ddd_index/).

### Statistical analyses

Data were analyzed using the statistical software package SPSS (Version 21.0; SPSS Inc., Chicago, IL, USA). Variables were presented as either mean (± SD) or frequency (%). Among the participant groups, categorical and continuous variables were compared using the chi-square test and one-way analysis of variance (ANOVA), respectively. A two-tailed difference of *p* < 0.05 was considered statistically significant.

We adopted Multivariate Analysis of Covariance (MANCOVA) to determine inter-group differences in BACS and UPSA-B performance after controlling for age, gender, and level of education. Bonferroni correction was used as a post-hoc test for correcting multiple comparisons. We adopted Pearson correlation coefficients to examine the relationships between the BACS composite scores and the UPSA-B domains among the four participant groups. Linear regression analysis was applied to determine the relationships between cognitive function, psychopathology, and antipsychotic agents among patients with schizophrenia.

## Results

### Characteristics

Table 1 summarizes the characteristics of the four participant groups: 63 schizophrenia patients (mean age: 41.6 years, 57.1% males), 55 MDD patients (mean age: 46.3 years, 32.7% males), 43 BD patients (mean age: 44.6 years, 51.2% males), and 92 healthy control subjects (mean age: 44.7 years, 47.8% males). The healthy control subjects had the highest level of education among the four groups. Compared with MDD and BD patients, schizophrenia patients had the youngest age of disease onset, the longest duration of illness, the lowest rate of antidepressant or benzodiazepine use, and the highest rate of being treated with antipsychotics.

**Table 1:** Characteristics of patients with schizophrenia, patients with major depressive disorder (MDD), patients with bipolar I disorders (BD), and healthy control subjects

	Schizophrenia (*n* = 63)	MDD (*n* = 55)	BD (*n* = 43)	Controls (*n* = 92)	Statistic value	*p*-value
Gender, n (%)					χ^2^ = 7.398	0.060
Male	36 (57.1)	18 (32.7)	22 (51.2)	44 (47.8)		
Female	27 (42.9)	37 (67.3)	21 (48.8)	48 (52.2)		
Age (years)	41.6 ± 8.9	46.3 ± 12.4	44.6 ± 12.3	44.7 ± 10.3	*F* = 1.988	0.116
Years of education	12.8 ± 2.8	13.0 ± 3.0	12.4 ± 2.7	14.6 ± 2.6	*F* = 9.347	< 0.001***
Age of onset (years)	25.5 ± 7.8	37.4 ± 12.3	32.9 ± 12.3	–	*F* = 18.368	< 0.001***
Duration of illness (years)	16.1 ± 9.1	8.9 ± 7.9	11.7 ± 7.7	–	*F* = 11.197	< 0.001***
Pharmacotherapy						
Antidepressant use, n (%)	5 (7.9)	41 (74.5)	11 (25.6)	–	χ^2^ = 59.443	< 0.001***
Antipsychotics use, n (%)	63 (100)	18 (32.7)	29 (67.4)	–	χ^2^ = 61.424	< 0.001***
Defined daily dose	1.0 ± 0.9	0.3 ± 0.3	0.7 ± 0.5		*F* = 6.696	0.002**
Olanzapine or clozapine	27 (42.9)	0 (0)	3 (10.3)		χ^2^ = 18.654	< 0.001***
Other antipsychotics	36 (57.1)	18 (100)	26 (89.7)			
Benzodiazepine use, n (%)	39 (61.9)	46 (83.6)	31 (72.1)	–	χ^2^ = 6.886	0.032*
Mood stabilizers use, n (%)	8 (12.7)	1 (1.8)	29 (67.4)	–	χ^2^ = 64.460	< 0.001***
Psychopathology assessments						
PANSS total scores	75.2 ± 19.0	–	–	–	–	–
Positive symptoms	17.1 ± 5.0	–	–	–		
Negative symptoms	19.2 ± 7.1	–	–	–		
General symptoms	38.9 ± 9.4	–	–	–		
YMRS total scores	–	–	3.4 ± 4.0	–	–	–
HAMD-17 items total scores	5.9 ± 5.2	7.5 ± 4.5	3.7 ± 3.1	–	*F* = 8.637	< 0.001***

Note: data are expressed as mean ± SD or n (%). *HAM-D* the 17-item Hamilton Depression Rating Scale, *PANSS* the Positive and Negative Syndrome Scale, *YMRS* the Young Mania Rating Scale. *p < 0.05, **p < 0.01, ***p < 0.001

### Profiles of cognitive function and functional capacity

**Fig. 1:**
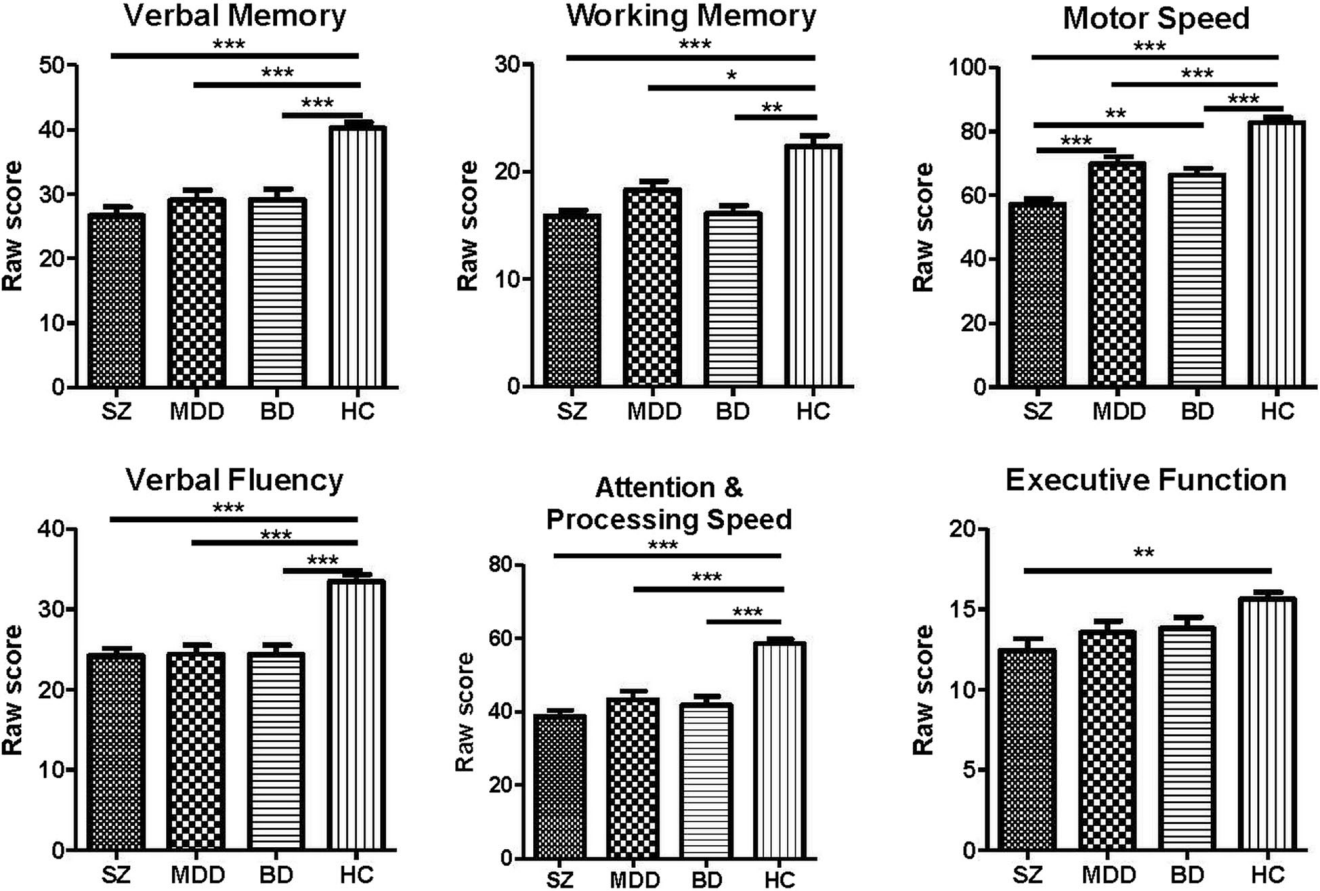
The six domains of the Brief Assessment of Cognition in Schizophrenia (BACS) across patients with schizophrenia (SZ), patients with major depressive disorder (MDD), patients with bipolar I disorders (BD), and healthy control subjects (HC). *p < 0.05, **p < 0.01, ***p < 0.001

After controlling for age, gender, and education (Fig. 1), compared to healthy controls, schizophrenia patients performed worse in all BACS subtests. Both the MDD and BD groups performed worse than the healthy control group in verbal memory, working memory, motor speed, verbal fluency, attention, and processing speed. Furthermore, schizophrenia patients had worse motor speed performance than both MDD and BD patients.

**Fig. 2:**
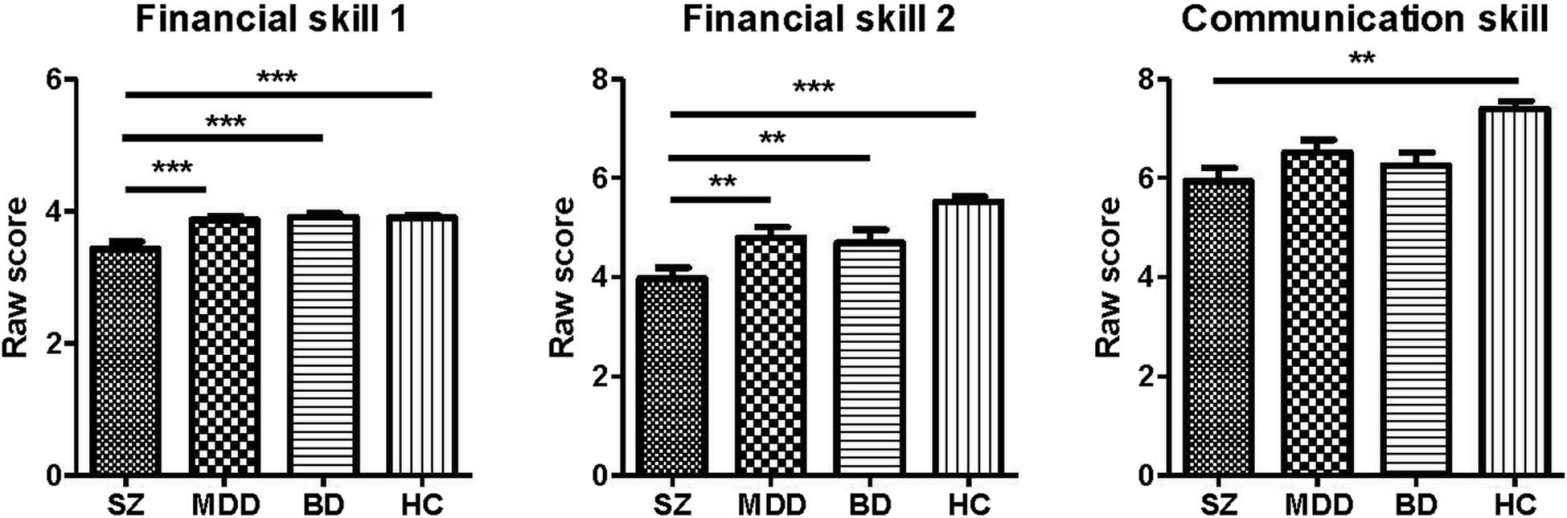
The three domains of the UCSD Performance-based Skills Assessment, Brief Version (UPSA-B) across patients with schizophrenia (SZ), patients with major depressive disorder (MDD), patients with bipolar I disorders (BD), and healthy control subjects (HC). *p < 0.05, **p < 0.01, ***p < 0.001

Regarding group differences in the UPSA-B (Fig. 2), schizophrenia patients performed worse in all three UPSA-B domains than the healthy controls. Patients with schizophrenia also performed worse than both the MDD patients and the BD patients in the financial portion (Financial skill 1 and Financial skill 2), but not in the communication domain.

### Relationships between BACS and UPSA-B

Table 2 shows the correlation coefficients between the BACS composite scores and the UPSA-B domains among the four participant groups. In the MDD group, the BACS composite score was positively correlated to all UPSA-B domains. In the schizophrenia and control groups, the BACS composite score was significantly correlated to Financial skill 2 and Communication skill. In the BD group, the BACS composite score was only correlated to Financial skill 2.

**Table 2:** Correlation between the BACS composite score and UPSA-B performance among patients with schizophrenia, patients with major depressive disorder (MDD), patients with bipolar I disorders (BD), and healthy control subjects

UPSA-B	Schizophrenia (*n* = 63)		MDD (*n* = 55)		BD (*n* = 43)		Controls (*n* = 98)	
	*r*	*p*-value	*r*	*p*-value	*r*	*p*-value	*r*	*p*-value
Financial skill 1	0.144	0.259	0.415	< 0.001***	0.114	0.467	0.170	0.094
Financial skill 2	0.425	0.001**	0.521	< 0.001***	0.368	0.015*	0.212	0.036*
Communication skill	0.429	< 0.001***	0.552	< 0.001***	0.258	0.095	0.226	0.025*

*p < 0.05, **p < 0.01, ***p < 0.001

### Cognitive performance, functional capacity, and psychopathology

We further examined the relationships between cognitive function, psychopathology, and antipsychotic agents among patients with schizophrenia (*n* = 63) using linear regression models. After controlling for age, gender, and education level (Additional file 1: Table S1), we observed a significant negative correlation between negative psychotic symptoms and verbal memory, verbal fluency, attention and processing speed, and executive function, as well as a significant negative correlation between positive psychotic symptoms and Financial Skill 2 in UPSA-B. However, we observed no significant correlation between antipsychotic properties/doses, cognitive profiles, and functional capacity.

## Discussion

The primary purpose of this study was to determine the differences in cognitive profiles and functional capacity among patients with schizophrenia, MDD, or BD and healthy controls. Our main findings have provided some new insights: (a) patients with major psychiatric disorders (schizophrenia, MDD, and BD) had poorer performance in BACS subtests than healthy subjects, except in the executive function domain; (b) schizophrenia patients had poorer performance of motor speed than patients with affective disorders; (c) schizophrenia patients had the worst performance of the financial portion of UPSA-B when compared to patients with affective disorders and healthy subjects; and (d) no significant difference was observed between patients with BD and MDD in either cognitive function or in functional capacity.

Our findings indicate that patients with major psychiatric disorders and healthy subjects had significantly different cognitive performances when assessed with BACS. Of the three patient groups, patients with schizophrenia displayed worse performance in motor speed than MDD patients and BD patients. Previous research has revealed that, compared with patients with mood disorders, those with schizophrenia may exhibit more impairments in various cognitive functions [48–50]. Our finding also agrees with the result that the cognitive deficit is more severe when a patient endures more psychosis rather than affective features, which suggests a continuum model of cognitive impairment in psychotic disorders [9]. Previous works have also agreed with the above findings that the motor speed of patients with schizophrenia was significantly worse than that of BD patients [51, 52]. As for the relationship between schizophrenia syndromes and cognitive deficit, our work supports that negative symptoms are associated with more severe cognitive dysfunction than positive symptoms [53]. Although the prescription strategy of antipsychotics may have a negative effect on cognitive function in schizophrenia patients [54, 55], other studies have shown inconsistent findings with no association [56] or positive effect [57]. In this study, we observed no significant correlation between motor speed and antipsychotic properties or dosages, as well as with other BACS subtests and functional capacity. Therefore, motor speed may have the potential to behave as the major cognition domain with the sensitivity to distinguish patients with schizophrenia from patients with affective disorders.

As for the functional capacity measured with the UPSA-B, schizophrenia patients performed worse than healthy controls in all three UPSA-B domains, as well as worse than both patients with MDD and those with BD in the financial portion. This finding agrees with those of previous studies, which have suggested that UPSA-B can effectively distinguish people with schizophrenia from those with affective disorders [31, 32]. Moreover, we observed no significant differences of the UPSA-B domains between MDD and BD patients, thus indicating that the performance of UPSA-B may have a ceiling effect [58]. We observed that BD patients had no difference on the UPSA-B compared with healthy controls, a finding that is consistent with the results of a previous study [33]. Patients with schizophrenia exhibited the worst cognitive performance and were easily identified from healthy controls. This result is in agreement with a prior study that reported that functional outcomes in BD tended to be better than those in schizophrenia [59]. But the disparity of daily function between patients with mood disorders in a euthymic state and healthy controls was not sufficiently significant to be discovered using the UPSA-B.

Previous studies have used BACS and UPSA-B to investigate the cognitive profiles and functional capacity of schizophrenia patients alone [21, 60, 61]. Two studies have suggested that the BACS was significantly associated with the daily function capacity measured by the UPSA-B [21, 60]. Sumiyoshi et al. [61] indicated that rating scores of the social function scale were more significantly correlated with individuals’ objective functional performance. This study is the first to simultaneously use the BACS (a cognitive assessment battery) and UPSA-B (functional capacity) to investigate the correlation between cognition and the three domains of functional capacity, not only in patients with schizophrenia but also in patients with affective disorders (MDD and BD). Among the four groups, the correlation coefficients between UPSA-B domains and BACS composite scores varied across the diagnoses. Composite measures coordinate several cognitive processes, and the scores are usually associated with functional ability [9]. We observed that BACS composite scores of MDD were moderate-to-strongly associated with all domains of UPSA-B. However, such a generalized correlation between cognition and functional capacity was diminished among patients with schizophrenia and BD. The finding of diverse relevance to various domains of functional capacity suggests that UPSA-B may target specific cognitive domains in different major psychiatric diagnoses. The result supports that combination with another endophenotyping measure while administrating BACS battery as an endophenotyping cognition assessment should be considered [9]. On the other hand, social cognition represents a primary predictor of functional outcome in schizophrenia and BD, serving as a mediating role between cognition and functioning [25]. The lack of social cognitive evaluation may reduce the significance of the results. Furthermore, the euthymic status of MDD and BD patients in this study also limited the interpretation. Therefore, the result should be cautiously generalized to the overall population.

This study has some limitations. First, the study observed cross-sectional findings rather than causal relationships. The measured cognitive profiles in this study may represent a cognitive state, which was not identical to a patient’s cognitive trait. Although all participants were evaluated at a relatively stable or euthymic state, the heterogeneity (i.e., partial/full remission; manic/depressive episode) of the patients may have affected the results. Second, the age, gender, and education levels among the patient groups and the healthy controls were not precisely matched. Third, the potential influence of the severity of clinical symptoms and categories and dosages of psychotropic drugs were not examined in this study as the healthy controls were drug-free and not assessed for disease characteristics. Therefore, we were unable to control for these variables in the statistical analyses. Fourth, several confounding factors that may alter cognition performance (e.g., cognition-related genes, premorbid function, intelligence quote, duration of illness, comorbidities, and tobacco use) were not included in this study. Finally, our sample size was not large, and the analysis lacks a replication sample. Our study’s findings should be verified in future studies using larger sample sizes.

## Conclusions

Considering overall performance, the BACS (a cognitive assessment battery) and the UPSA-B (functional capacity) characterize different endophenotyping profiles in the aforementioned four groups of participants examined in this study. Our results support a divergence between the two constructs of functioning and their underlying components and highlight the need to target both dimensions in patients with major psychiatric disorders.

## Supplementary information

**Additional file 1: Table S1.** Relationships between cognitive function, psychopathology and antipsychotic agents among patients with schizophrenia (*n* = 63).

## Data Availability

Specific data sets used and/or analysed during the current study are available from the corresponding author on reasonable request.

## Abbreviations

ANOVA: One-way analysis of variance
BACS: The Brief Assessment of Cognition in Schizophrenia
BD: Bipolar I disorder
DSM-IV-TR: The Diagnostic and Statistical Manual of Mental Disorders, Fourth Edition
HAMD-17: The 17-item Hamilton Depression Rating Scale
KCGMH: Kaohsiung Chang Gung Memorial Hospital
MANCOVA: Multivariate Analysis of Covariance
MDD: Major depressive disorder
PANSS: The Positive and Negative Syndrome Scale
UPSA-B: The UCSD Performance-based Skills Assessment, Brief Version
YMRS: The Young Mania Rating Scale
