# Supplementary material for: Defining cognitive and functional profiles in schizophrenia and affective disorders
Source: BMC Psychiatry. 2020 Jan 31;20:39. doi: 10.1186/s12888-020-2459-y (PMC6995055; doi:10.1186/s12888-020-2459-y)
Supplement: Supplementary file 1 — Additional file 1: Table S1. Relationships between cognitive function, psychopathology and antipsychotic agents among patients with schizophrenia (n = 63). [file 12888_2020_2459_MOESM1_ESM.doc]

**Additional file 1: Table S1.** Relationships between cognitive function, psychopathology and antipsychotic agents among patients with schizophrenia (n=63)

|  | | |  | | | |  | | | | **BACS** | | | | | |  | | |  | | | | |  | **UPSA-B** | | | | | | | | | |  |
| --- | --- | --- | --- | --- | --- | --- | --- | --- | --- | --- | --- | --- | --- | --- | --- | --- | --- | --- | --- | --- | --- | --- | --- | --- | --- | --- | --- | --- | --- | --- | --- | --- | --- | --- | --- | --- |
| **Variables** | | Verbal memory | | | | Working memory | | | | Motor speed | | | | Verbal fluency | | Attention and processing speed | | | Executive  function | | | |  | Financial  skill 1 | | | | | Financial  skill 2 | | | | Communication skill | | |  |
|  | |  | |  | |  | |  | |  | |  | |  |  |  | |  |  | | |  |  |  | | | |  |  | |  |  | |  | |  |
| Age | 0.06 (-0.32, 0.43) | | | | 0.37(0.00, 0.73) | | | | -0.07(-0.50, 0.36) | | | | 0.09(-0.18, 0.35) | | | 0.20(-0.09, 0.50) | | | | | -0.15(-0.88, 0.58) | | | | | | -0.04(-0.08, 0.01) | | | 0.02(-0.02, 0.06) | | | | | 0.00(-0.03, 0.04) | |
| Sex | -4.90 (-11.52, 1.71) | | | | -2.18(-8.61, 4.25) | | | | **-16.11(-23.64, -8.57)***** | | | | 1.36(-3.23, 5.95) | | | -4.68(-9.90, 0.55) | | | | | **-17.41(-30.23, -4.59)**** | | | | | | 0.40(-0.39, 1.19) | | | -0.03(-0.65, 0.60) | | | | | -0.20(-0.84, 0.43) | |
| Education | 0.79(-0.37, 1.96) | | | | 0.42 (-0.72, 1.55) | | | | 0.79(-0.54, 2.13) | | | | **1.11(0.30, 1.92)**** | | | **0.97(0.04, 1.89)*** | | | | | **2.41(0.15, 4.68)*** | | | | | | 0.00(-0.14, 0.14) | | | -0.02(-0.13, 0.09) | | | | | -0.01(-0.13, 0.10) | |
| Antipsychotics | -1.68(-8.43, 5.08) | | | | -2.50 (-9.07, 4.07) | | | | 2.60(-5.09, 10.30) | | | | -3.34(-8.03, 1.34) | | | -4.09(-9.43, 1.25) | | | | | 1.35(-11.74, 14.44) | | | | | | 0.33(-0.48, 1.13) | | | -0.24(-0.88, 0.40) | | | | | 0.40(-0.26, 1.05) | |
| DDD | 0.43(-3.20, 4.06) | | | | 0.15 (-3.38, 3.68) | | | | -0.81(-4.95, 3.32) | | | | -0.78(-3.30, 1.74) | | | -0.43(-3.30, 2.44) | | | | | -1.80(-8.84, 5.23) | | | | | | 0.17(-0.27, 0.60) | | | 0.08(-0.27, 0.42) | | | | | 0.01(-0.34, 0.36) | |
| PANSS-P | 0.17(-0.68, 1.02) | | | | -0.55(-1.38, 0.28) | | | | 0.58 (-0.39, 1.55) | | | | 0.53(-0.06, 1.12) | | | 0.47(-0.20, 1.14) | | | | | -0.86(-2.51, 0.79) | | | | | | -0.02(-0.12, 0.08) | | | **-0.09(-0.17, -0.01)*** | | | | | -0.02(-0.10, 0.07) | |
| PANSS-N | **-0.82(-1.58, -0.05)*** | | | | -0.65(-1.40, 0.09) | | | | -0.14(-1.01, 0.73) | | | | **-0.68(-1.21, -0.15)*** | | | **-0.83(-1.44, -0.23)**** | | | | | **-1.51(-2.99, -0.02)*** | | | | | | -0.04(-0.13, 0.05) | | | -0.06(-0.14, 0.01) | | | | | -0.07(-0.15, 0.00) | |
| PANSS-G | 0.36(-0.33, 1.05) | | | | 0.49 (-0.18, 1.17) | | | | -0.35(-1.14, 0.44) | | | | 0.22(-0.26, 0.70) | | | 0.03(-0.51, 0.58) | | | | | 0.84(-0.50, 2.18) | | | | | | 0.00(-0.08, 0.08) | | | 0.06(-0.01, 0.12) | | | | | 0.04(-0.03, 0.11) | |
| **Model Summary** | | | | |  | | | |  | | | |  | | |  | | | | |  | | | | | |  | | |  | | | | |  | |
| R^2^ | 0.345 | | | | 0.275 | | | | 0.222 | | | | 0.366 | | | 0.575 | | | | | 0.393 | | | | | | 0.296 | | | 0.309 | | | | | 0.292 | |
| F | 3.687 | | | | 2.657 | | | | 1.998 | | | | 4.050 | | | 9.478 | | | | | 4.527 | | | | | | 2.937 | | | 3.127 | | | | | 2.886 | |
| p-value | 0.002 | | | | 0.015 | | | | 0.063 | | | | 0.001 | | | < 0.001 | | | | | < 0.001 | | | | | | 0.008 | | | 0.005 | | | | | 0.009. | |

Note: BACS, the Brief Assessment of Cognition in Schizophrenia; DDD, defined daily dose; PANSS-P, Positive and Negative Syndrome Scale positive score; PANSS-N,

Positive and Negative Syndrome Scale negative score; PANSS-G, Positive and Negative Syndrome Scale general psychopathology score; UPSA-B, the UCSD Performance –

based Skills Assessment, Brief Version.

The data represented here is B (95% Confidence Interval). **p<0.05, **p<0.01, ***p<0.001*
